# Structural basis for polyspecificity in the POT family of proton-coupled oligopeptide transporters

**DOI:** 10.15252/embr.201338403

**Published:** 2014-06-10

**Authors:** Joseph A Lyons, Joanne L Parker, Nicolae Solcan, Alette Brinth, Dianfan Li, Syed TA Shah, Martin Caffrey, Simon Newstead

**Affiliations:** 1 Schools of Medicine and Biochemistry & Immunology, Trinity College DublinDublin, Ireland; 2 Department of Biochemistry, University of OxfordOxford, UK; † Department of Molecular Biology and Genetics, Aarhus UniversityAarhus, Denmark

**Keywords:** crystallography, major facilitator superfamily, membrane protein, peptide binding site, POT/PTR family

## Abstract

An enigma in the field of peptide transport is the structural basis for ligand promiscuity, as exemplified by PepT1, the mammalian plasma membrane peptide transporter. Here, we present crystal structures of di- and tripeptide-bound complexes of a bacterial homologue of PepT1, which reveal at least two mechanisms for peptide recognition that operate within a single, centrally located binding site. The dipeptide was orientated laterally in the binding site, whereas the tripeptide revealed an alternative vertical binding mode. The co-crystal structures combined with functional studies reveal that biochemically distinct peptide-binding sites likely operate within the POT/PTR family of proton-coupled symporters and suggest that transport promiscuity has arisen in part through the ability of the binding site to accommodate peptides in multiple orientations for transport.

## Introduction

Membrane transporters involved in nutrient uptake are usually substrate specific, recognizing and transporting only chemically very similar ligands across the membrane 1. However, a number of mammalian nutrient transporters also recognize and transport a variety of drug molecules, in addition to their intended nutrients, with important implications for drug pharmacokinetics and drug–drug interactions 2. These include the organic anion-transporting polypeptides (OATPs), the organic anion and cation transporters (OAT and OCT) and the proton-coupled peptide transporters (POT/PTR family) 3,4. The molecular basis for this promiscuity or polyspecificity is an important biochemical question that must be addressed if their role in drug transport is to be understood and usefully exploited.

The human POT family transporters, PepT1 (SLC15A1) and PepT2 (SLC15A2), are responsible for peptide transport across the plasma membrane and have evolved one of the most promiscuous binding sites in biology, capable of recognizing and transporting over 8,000 different di- and tripeptide ligands 5. They are of increasing pharmaceutical importance as they recognize and transport a growing library of antibiotics, antiviral and anticancer molecules 6. The POT (or PTR) family belongs to the major facilitator superfamily (MFS) of secondary active transporters that includes the OATPs, OATs and OCTs 7–9. The POT/PTR family contains a number of conserved sequence motifs (Supplementary Fig S1) and a conserved architecture consisting of 12 or 14 transmembrane α-helices arranged into N (H1–6)- and C-terminal (H7–12) bundles 3,10–12. A centrally located peptide-binding site is highly conserved between prokaryote and eukaryote members, with both operating via similar transport mechanisms 13. Conserved pairs of salt bridge interactions coordinate structural rearrangements that lead to the vectorial co-transport of peptides and protons across the membrane 3,11.

A key question we sought to address was how one binding site could specifically recognize and transport such a large number of different ligands, while retaining specificity for peptides only two or three amino acids long. To address this question, we determined high-resolution crystal structures of a bacterial homologue to the mammalian PepT1 protein from *Streptococcus thermophilus*, PepT_St_ in complex with physiologically relevant di- and tripeptides in addition to a peptide-free apo form (Table 1). POT family transporters are understood to transport both di- and tripeptides 13. However, for PepT_St_ and other bacterial members of the family, there exists some selectivity for different di- and tripeptides 14,15. We previously showed that PepT_St_ displays broad specificity for dipeptides, being able to transport a range of charged and hydrophobic peptides with IC_50_ values in the region of 5–400 μM 3. Tripeptides are also transported, with tri-alanine being the best peptide tested to date with an IC_50_ value comparable to that of the dipeptides (Supplementary Fig S2). PepT_St_ therefore represents a good model with which to study the structural basis for substrate selectively within the POT family. The current structures and functional data reveal a binding site that can accommodate peptides in different orientations that helps to explain the broad substrate specificity characteristic of this pharmaceutically relevant transporter family.

**Table 1:** Data collection and refinement statistics

	PepT_St_ apo	PepT_St_ AAA^c^	PepT_St_ AF
**Data collection**			
Space group	C222_1_	C222_1_	C222_1_
Cell dimensions			
*a*, *b*, *c* (Å)	102.2, 110.2, 111.0	103.4, 110.7, 110.6	102.1, 110.3, 110.7
α, β, γ (°)	90.0, 90.0, 90.0	90.0, 90.0, 90.0	90.0, 90.0, 90.0
Wavelength (Å)	0.9686	1.0332	0.9686
Resolution (Å)^a^	39.1–2.35 (2.48–2.35)	75.56–2.52 (2.59–2.52)	51.03–2.47 (2.53–2.47)
CC1/2(%)^b^	98.6 (63.4)	99.6 (74.6)	99.8 (53.0)
*R*_merge_	15.8 (71.0)	8.0 (56.8)	7.3 (64.0)
*R*_pim_	6.1 (37.7)	5.2 (37.0)	4.6 (40.8)
*I*/σ*I*	8.3 (2.2)	10.4 (2.2)	10.9 (2.0)
Completeness (%)	99.3 (98.0)	98.7 (99.3)	97.9 (99.4)
Redundancy	6.0 (4.1)	3.2 (3.2)	4.1 (4.2)
Number of crystals	7	1	1
**Refinement**			
Resolution (Å)	39.1–2.35	75.56–2.52	51.04–2.47
Number of reflections	26,224	21,332	22,139
*R*_work/_*R*_free_	21.3/24.2	19.8/24.2	22.2/26.6
Number of atoms			
Protein	3,440	3,582	3,321
Ligand	N/A	16	17
Lipid	198	132	132
Ions	5	5	5
Water	54	45	38
B-factors (Å^2^)			
Protein	38.4	49.8	39.7
Ligand	N/A	75.9^c^	62.8
Lipid	54.8	61.4	54.0
Ions	56.4	69.6	61.7
Water	34.2	44.1	36.9
R.m.s deviations			
Bond lengths (Å)	0.006	0.006	0.006
Bond angles (°)	0.913	0.915	0.879
Ramachandran statistics favoured/outliers (%)	99.0/0	98.3/0.2	99.0/0/0

a Highest resolution shell is shown in parenthesis.

b Percentage of correlation between intensities from random half-datasets, as given by XDS.

c PepT_St_ tri-Ala complex was deposited with the carboxy terminus of the tripeptide oriented towards the extracellular side of the membrane.

## Results and Discussion

### Physiological dipeptide complex

**Figure 1:**
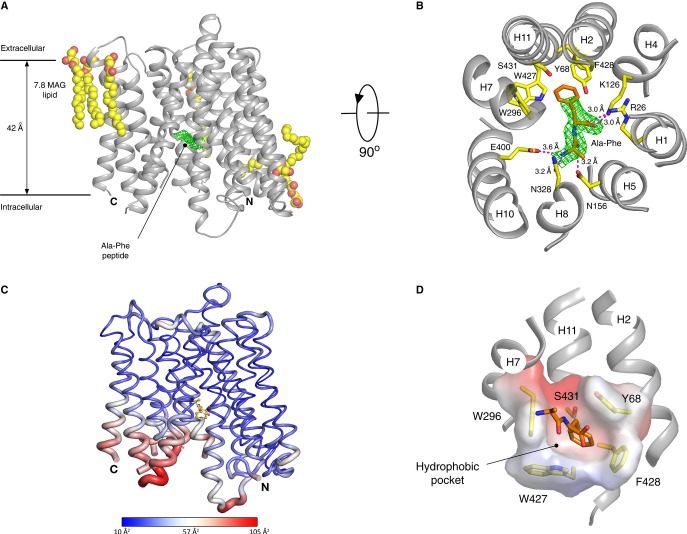
Structure of PepT_St_ with Ala-Phe dipeptide A Side on view of PepT_St_ with the 7.8 MAG lipid shown in spheres. The m*F*_o_-D*F*_c_ difference electron density map (green) is shown, contoured at 3.0 σ. B Extracellular view of the peptide-binding site with hydrogen bonds as dashed lines. C Flexibility in the C-terminal domain indicated by the atomic displacement-coded putty thickness and colour gradient from blue (low disorder) to red (high disorder). The average atomic displacement parameter for the protein is 39.7 Å^2^. D View into the conserved hydrophobic pocket accommodating the ligand’s phenylalanine side chain in the peptide.

A co-crystal structure with the natural dipeptide l-Ala-l-Phe (Ala-Phe) was obtained using the *in meso* crystallization method 16 and refined to a maximum resolution of 2.5 Å (Materials and Methods, Table 1 and Supplementary Fig S3). The peptide was clearly visible in the m*F*_o_-D*F*_c_ difference electron density maps and sits laterally in the binding site (Fig 1A and B). The Ala-Phe peptide is held by electrostatic interactions between its amino and carboxy termini and side chains extending from both the N- and C-terminal bundles (Fig 1B). This binding mode is similar to the previous POT family homologues co-crystallized with the antibacterial phosphonopeptide, alafosfalin 11,12 (Supplementary Fig S4). The peptide amino terminus interacts with a conserved glutamate (Glu400) on H10 and through a hydrogen bond to an asparagine (Asn328) on H8. These residues are essential for binding and transport of peptides in PepT_St_ 3. The amide nitrogen does not interact with the binding site, consistent with previous predictions that only the carbonyl group of the peptide bond is recognized in PepT1. The carbonyl group of the peptide bond is coordinated to a conserved asparagine (Asn156) on H5, forming a ligand-coordinated bridge between the two six-helix bundles. The C-terminal bundle in PepT_St_ appears to be more flexible than the opposing N-terminal bundle, as evidenced by the atomic displacement parameters (Fig 1C), and consistent with this region undergoing more structural change during transport.

Interestingly, the phenyl ring of the peptide is accommodated in a previously unobserved hydrophobic pocket (pocket 1) approximately 10 × 10 × 3 Å in size and formed by side chains from H2 (Tyr68), H7 (Trp296) and H11 (Trp427, Phe428, Ser431) (Fig 1D). The presence of such a pocket was predicted previously based on transport and modelling data in PepT1 7. The side chain of Tyr68 is positioned 3.9 Å away, making a pi–pi stacking interaction with the phenyl group. The close interaction of Tyr68 with the dipeptide is also consistent with our previous study on PepT_St_ showing that Tyr68 plays an important role in determining dipeptide specificity 3. At the N-terminal end of the ligand, another elongated cavity, pocket 2, with dimensions approximately 16 × 7 × 11 Å, is present that may accommodate a larger side chain at this position (Supplementary Fig S5). The C-terminus of the ligand interacts with both a conserved arginine on H1 (Arg26) and a lysine on H4 (Lys126). High-affinity ligands of PepT1 share a common feature of having their N- and C-terminal groups separated by approximately 6 Å 17. Our structure suggests this requirement is due to the positioning of these groups between the previously observed dipole 3,10 within the binding site to orientate peptides laterally (Fig 1B). Tripeptides by contrast are considerably longer than dipeptides, approximately 10 Å fully extended. Based on the results to date, it was clear that extended tripeptides could not be accommodated in the same way as the Ala-Phe ligand. We speculated that tripeptides would therefore be recognized differently, with important implications for understanding the transport of β-lactam antibiotics, which are similar to tripeptides in size (Supplementary Fig S6).

### Peptides can also bind in a vertical orientation

**Figure 2:**
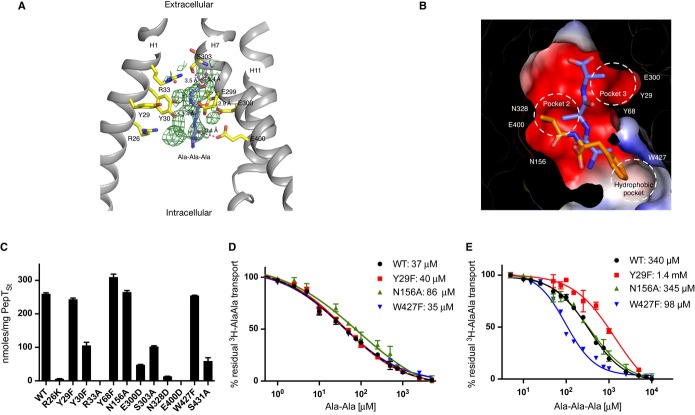
Tri-alanine binds to PepT_St_ in a vertical orientation A View of the binding site in the plane of the membrane, showing the m*F*_o_-D*F*_c_ difference electron density map, contoured at 3.0 σ. Hydrogen bonds are shown as dashed lines. In this panel, the peptide was modelled with the C-terminus orientated towards the apex of the cavity. B Electrostatic surface representation of the binding site in the Ala-Phe complex structure with both peptides superimposed illustrating the relative orientations. The side chains of key residues involved in peptide binding are outlined. C Effect of binding site mutations on proton-driven di-alanine uptake in liposomes. D IC_50_ competition curves for di-alanine. E IC_50_ competition curves for tri-alanine showing residual uptake of [^3^H]-di-Ala peptide normalized to WT. Data information: Error bars indicate the standard deviation from three independent experiments.

A new crystal structure in complex with the tripeptide l-Ala-l-Ala-l-Ala (tri-Ala), which was the only tested tripeptide to inhibit di-Ala transport (Supplementary Fig S2), was obtained and refined to a final resolution of 2.5 Å (Materials and Methods and Table 1). Careful analyses of the electron density maps, including the calculation of simulated annealing OMIT maps and averaged kick maps 18, clearly showed the tri-Ala peptide sitting in a vertical orientation (Fig 2A and Supplementary Fig S7). The data, however, do not allow us to establish how the peptide is orientated in the binding site, with its C-terminus facing towards the cytoplasmic or periplasmic space. This ambiguity arises due to ill-defined electron density that likely reflects low occupancy of the peptide in the binding site. For the purpose of the discussion that follows, we focus on the peptide modelled with the C-terminus facing the periplasm. This choice is based on functional data for peptide recognition in PepT1 that suggests the C-terminus of the peptide interacts with residues at the extracellular entrance to the binding site 19. Compared to the Ala-Phe complex, tri-Ala makes far fewer interactions with the protein. This explains, in part, the disparate affinities of PepT_St_ for di- and tripeptides, and unlike the dipeptide, the tri-Ala may be protonated as it sits within hydrogen bonding distance of both the backbone carbonyl groups of Glu299 and Glu300 on H7 and the backbone amide nitrogen of Ser303. The tripeptide sits in an elongated cavity formed by H1 (Tyr30), H5 (Asn156), H7 (Glu299, 300) and H8 (Gln325, Asn328) and above the alanine side chain observed in the Ala-Phe complex (Fig 2B). The extracellular end of the cavity is sealed by the packing together of H1 and H7, which form the extracellular gate in the POT family 10. Another noticeable difference is the presence of a third smaller cavity (pocket 3) that opens up opposite the third side chain in the tri-Ala peptide as a result of the less compact structure adopted by PepT_St_ in the tri-Ala complex compared to the Ala-Phe complex. The side chains of Glu299 and Glu300 are within hydrogen bond distance to the carbonyl of the C-terminal peptide bond. Glu299, however, is not conserved within the wider POT family; it is a phenylalanine in PepT1 for example (Supplementary Fig S1). It seems reasonable that the interaction with tripeptides in this region will differ between homologues, further explaining the differences observed in substrate specificity with respect to di- and tripeptides discussed above 14. The nitrogen of the C-terminal peptide bond in tri-Ala peptide interacts with the side chain of Tyr30. This aromatic residue forms part of the highly conserved E^22^xxERFxYY motif on H1 and was previously shown to play a role in proton binding 3,11. The N-terminal peptide carbonyl is within hydrogen bond distance to Glu400. As modelled, the amino terminus of the tri-Ala peptide makes no interaction within the binding site. This is consistent with previous studies using PepT2 where the C-terminus of tripeptides was shown to be more important in tripeptide versus dipeptide transport (reviewed in 20).

### Binding modes are functionally distinct

The PepT_St_ complex structures in this study show that although the di- and tripeptides are positioned differently in the binding site, the first two side chains of both ligands are similarly orientated (Fig 2B). If PepT_St_ can bind peptides in these two orientations, we should be able to identify mutations that selectively affect only peptides recognized in these two modes. To investigate this possibility, residues observed interacting with the two peptides were mutated and functionally tested using a proton-driven peptide uptake assay 3 (Fig 2C). Aside from Tyr68, already discussed, three further residues were identified that interacted with the peptides but that did not result in reduced overall transport upon substitution; they included Tyr29Phe, Asn156Ala and Trp427Phe. In the di-Ala uptake study, these variants showed similar IC_50_ values, with one exception; Asn156Ala showed a small but significant decrease in affinity (86 μM compared to 37 μM for the WT transporter) (Fig 2D). This result is consistent with complex structure where Asn156 forms a hydrogen bond to the carbonyl group of the Ala-Phe peptide (Fig 1B and D). By comparison, the same Asn156Ala variant had no effect on tri-Ala competition (Fig 2E), consistent with the complex structure where Asn156 does not interact with the tri-Ala peptide (Fig 2A and Supplementary Fig S7). However, Tyr29Phe did show reduced competition (IC_50_ of 1.4 mM versus 340 μM for the WT) consistent with both Tyr30 and Tyr29 being important in tripeptide uptake 3. Interestingly, Trp427Phe showed increased affinity for tri-Ala compared to WT (IC_50_ 98 μM). Our structures do not provide an obvious explanation as to why reducing the size of this side chain would result in an increased affinity for tri-Ala peptide, but as discussed below this may indicate that other binding modes for tri-Ala exist within the binding site that are not revealed by our structures. However, the observation that different binding site mutants differentially affect substrate specificity further highlights the likelihood of multiple binding modes.

### Intracellular gate linked to formation of the hydrophobic pocket

**Figure 3:**
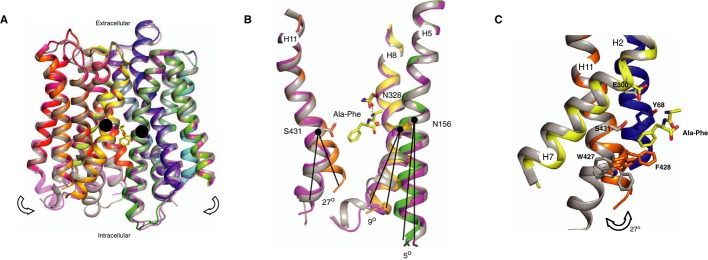
Conformational changes in PepT_S__t_ upon Ala-Phe binding A Three states of PepT_S__t_ are shown superimposed; Apo (grey), tri-Ala bound (purple) and Ala-Phe bound (coloured from the N-terminus blue to C-terminus red). The arrows indicate the major structural change observed between the crystal structures, and black dots identify the main hinge points in the N- and C-terminal bundles, respectively. B Structural comparison between the three structures reveals that in the Ala-Phe complex helices H5, H8 and H11 close in around the peptide. The side chains of two conserved asparagines make hydrogen bonds to the Ala-Phe peptide at the hinge points in the helices H5 (N156) and H8 (N328). C Zoomed in view of H11 showing the formation of the hydrophobic pocket around the phenylalanine side chain of the Ala-Phe peptide (coloured helices) and the dissolution of the pocket upon adopting the more open structure observed in the Apo structure (grey).

Comparing the structures of PepT_St_ in the peptide-bound states with a high-resolution peptide-free apo state determined to 2.35 Å (Materials and Methods and Table 1) reveals that the Ala-Phe complex assumes a more compact structure (Fig 3A). The movement appears localized to the C-terminal bundle and occurs following intra-helical bending at residues Ser431 (H11), Asn328 (H8) and to a lesser extent at Asn156 (H5), the latter two side chains making direct H-bond interactions with the Ala-Phe peptide (Figs 1D and 3B). This is consistent with our finding that mutation of either Asn328 or Ser431 severely reduced transport (Fig 2C). The cytoplasmic ends of H10 and H11 form an intracellular gate with a hinge region located around Gly407 (H10) and Trp427 (H11) 3. The structure of the Ala-Phe complex suggests that the closing of this gate around the dipeptide results in the formation of the hydrophobic pocket, which forms to accommodate the bulky phenyl side chain of the ligand (Fig 3C). These observations are consistent with an induced fit mechanism within the POT/PTR family with peptide binding facilitating structural rearrangement of the TM helices at sites of interaction with the peptide ligand.

### A multi-mode transport model for the POT family

**Figure 4:**
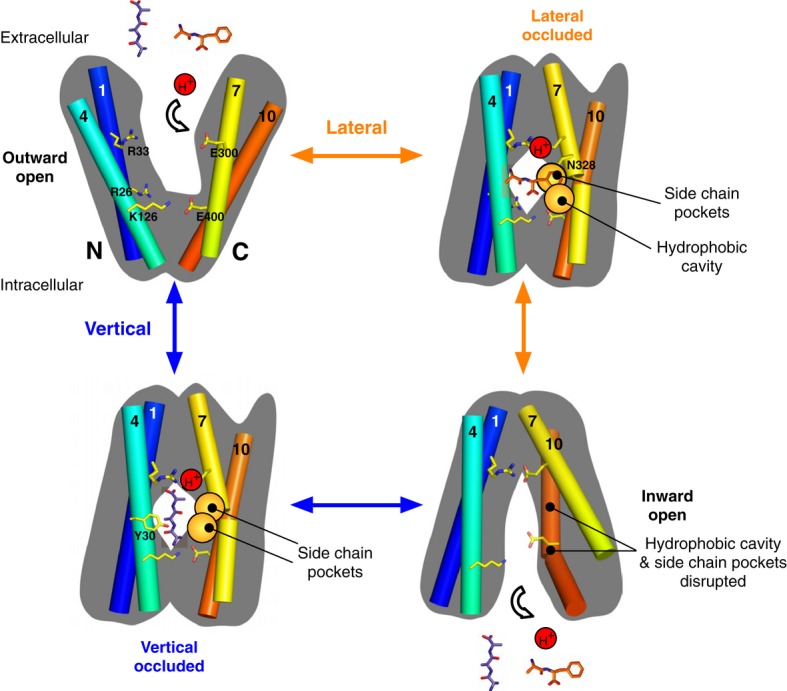
A multi-site model for peptide transport in the POT family In the outward-open state, the transporter will accept peptides and accommodate them in preferred orientations. This study has revealed two modes of binding, a lateral mode observed for the Ala-Phe peptide and a vertical mode for the tri-Ala peptide. In both cases, peptide and proton binding will drive the transporter to the occluded state, where the extracellular gate (H1-H7) is closed, stabilized by a salt bridge between Arg33 and Glu300 and the intracellular gate (H4-H10) salt bridge between Lys126 and Glu400 is disrupted 3. The structural transition required to drive reorientation of the binding site is potentially the result of proton binding to/release from Glu300 11. Peptide release from the transporter will occur following rearrangement of the binding site to the inward-open state characterized by a disrupted hydrophobic pocket and concomitant proton release into the cytoplasm.

This study reveals that polyspecificity in the POT family is likely to have arisen in part through the evolution of a binding site that can accommodate peptide ligands in at least two different binding modes. Further, the binding site can co-opt the same or similar pockets (hydrophobic, pockets 2 and 3) to accommodate the side chains, linking their formation and dissolution with the different states in the transport cycle to facilitate peptide recognition and release (Fig 4). It has been suggested that both LacY, the lactose permease and VMAT2, the vesicular monoamine transporter, facilitate transport via an induced fit mechanism 21,22. The comparison between the structures presented here suggests a similar mechanism may operate within the POT/PTR family. If so, it could play an important role in driving the structural changes required for peptide and drug transport. However, our data do not exclude the possibility that other binding modes exist for peptides. Indeed, it is plausible that the promiscuity of these transporters arises in large part due to their ability to accommodate peptides in multiple orientations and that this study reveals two of perhaps many different ways peptides can interact with these transporters.

## Materials and Methods

### Protein production and purification

Wild-type and mutant PepT_St_ were purified to homogeneity as described previously 3.

### Crystallization

The protein-laden mesophase was prepared by homogenizing 7.8 monoacylglycerol and 10 mg/ml protein solution in a 1:1 ratio by weight using a coupled syringe mixing device at 20°C 23. Crystallization trials were carried out at 20°C in 96-well glass sandwich plates with 50 nl mesophase and 0.8 μl precipitant solution using an *in meso* robot 24. For apo-PepT_St_, crystallization solutions consisted of 16–23% (v/v) polyethylene glycol 400, 0.1 M HEPES pH 7.0 and 0.15–0.55 M NH_4_H_2_PO_4_. For PepT_St_-peptide complexes, the protein was incubated with 10 mM peptide for 1 h on ice. Crystallization was carried out as described above with the screen supplemented with 10 mM peptide. 3-D pyramidal crystals grew to a maximum size of 40 × 40 × 40 μm^3^ in 3–5 days (Supplementary Fig S2). Wells were opened using a tungsten–carbide glasscutter, and the crystals were harvested using 30–50 μm micromounts (MiTeGen) 25. Crystals were snap-cooled directly in liquid nitrogen.

### Data collection and processing

X-ray diffraction data were collected on the 23-ID-B beamline (GM/CA-CAT) at the Advanced Photon Source (APS), Argonne, IL, USA, and the I24 beamline at the Diamond Light Source (DLS), Oxford, UK. Data were acquired using a 10-μm minibeam at GM/CA-CAT 26, while a 10-μm microfocus beam was used at DLS. Oscillation data were measured in 1.0° frames with 1–2 s exposures using a 1× or 10× attenuated beam at GM/CA-CAT or 0.2° frames with 0.2 s exposures at DLS. All data were initially reduced in xia2 27 using XDS 28, XSCALE and AIMLESS 29,30 (Table 1). For the apo-PepT_St_ data set, the data reduction strategy involved combining a complete medium resolution (3.0 Å) data set recorded from a single crystal using a 10× attenuated X-ray beam with six high-resolution (up to 2.3 Å) 20° wedges of data collected from multiple crystals. Data for the PepT_St_/peptide co-crystals were collected from a single crystal and processed in XDS, XSCALE and AIMLESS.

### Structure solution and refinement

Molecular replacement search models were prepared from the inward-open PepT_St_ model (PDB ID: 4APS) pruned of all side chains and non-protein atoms using Chainsaw 31. Initial phases were obtained by MR using Phaser 32. Iterative rounds of structure refinement were performed in PHENIX 33. The structural model was revised in real space with the program COOT 34 using sigma-A-weighted 2*F*_o_-*F*_c_ and m*F*_o_-D*F*_c_ electron density maps. The geometric quality of the model was assessed with MolProbity 35. Structures of ligands, lipids and water molecules were determined and refined.

### Peptides

For the Ala-Phe and tri-Ala PepT_St_ complexes, the respective peptide molecules were identified in unbiased m*F*_o_*-*D*F*_c_ difference electron density maps, standard OMIT 36 maps and average kick maps 18.

### Transport assays

PepT_St_ was reconstituted into *Escherichia coli* total lipids with egg PC liposomes and assayed using a proton-driven system as previously described 3.
