# Supplementary material for: Structural basis for polyspecificity in the POT family of proton-coupled oligopeptide transporters
Source: EMBO Rep. 2014 Jun 10;15(8):886–93. doi: 10.15252/embr.201338403 (PMC4149780; doi:10.15252/embr.201338403)
Supplement: Supplementary file 1 [file embr0015-0886-sd1.pdf]

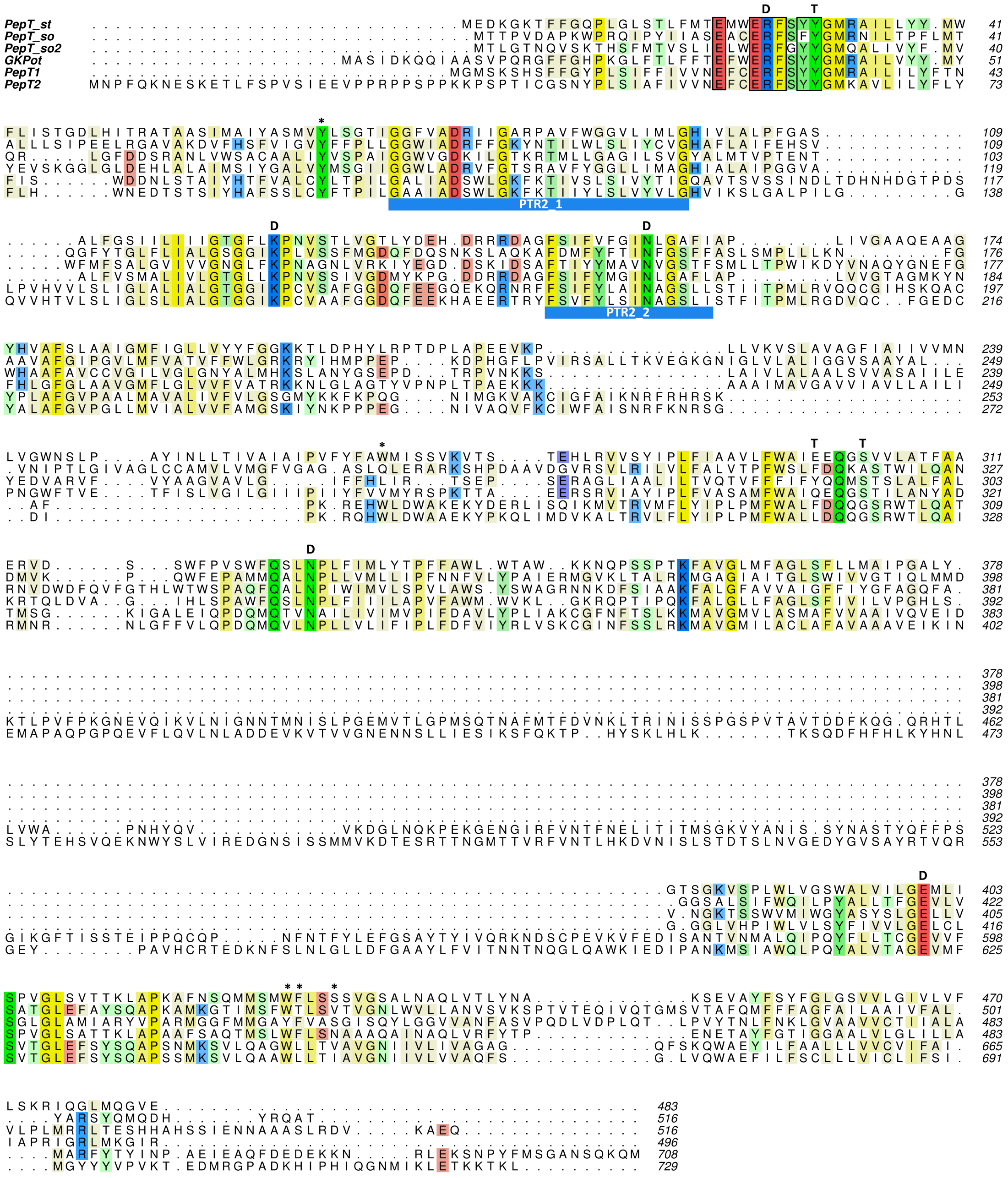

Figure S1. Sequence alignments for the current crystal structures of POT family members and their human PepT1 and PepT2 homologues. Color-coded based on amino acid type (acidic = red, basic = blue, neutral = yellow) and conservation. Residues involved in di-peptide and tri-peptide interaction and in hydrophobic pocket formation in PepT$_{St}$ are marked D, T and *, respectively. Residues in the ExxERFxYY motif are enclosed in black boxes; Peptide TRansporter (PTR) family signature motifs, PTR2_1 and PTR2_2 are indicated.
