# Supplementary material for: Structural basis for polyspecificity in the POT family of proton-coupled oligopeptide transporters
Source: EMBO Rep. 2014 Jun 10;15(8):886–93. doi: 10.15252/embr.201338403 (PMC4149780; doi:10.15252/embr.201338403)
Supplement: Supplementary file 2 [file embr0015-0886-sd2.pdf]

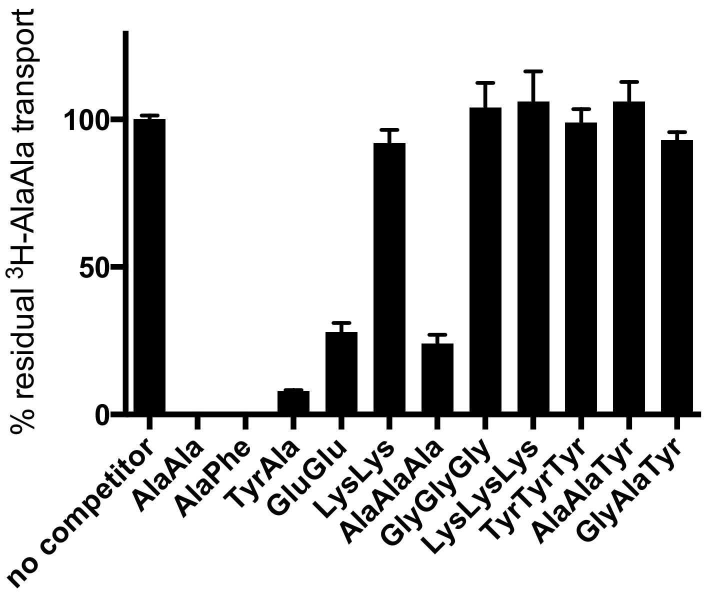

Figure S2. Substrate specificity profile for PepT$_{St}$. PepT$_{St}$ displays an overall preference for di-peptides but still recognizes tri-alanine with similar affinity to Glu-Glu and better than Lys-Lys. Larger tri-peptides and tri-glycine are not able to compete effectively against the reporter peptide ^{3}H-di-alanine. '% residual transport' refers to competition for ^{3}H-Ala-Ala uptake in the presence of 10 mM of the cold peptides indicated on the X-axis as a percentage of 'no competitor' peptide in a reconstituted proton driven uptake assay. Error bars indicate the standard deviation from three independent experiments.
