# Supplementary material for: Structural basis for polyspecificity in the POT family of proton-coupled oligopeptide transporters
Source: EMBO Rep. 2014 Jun 10;15(8):886–93. doi: 10.15252/embr.201338403 (PMC4149780; doi:10.15252/embr.201338403)
Supplement: Supplementary file 3 [file embr0015-0886-sd3.pdf]

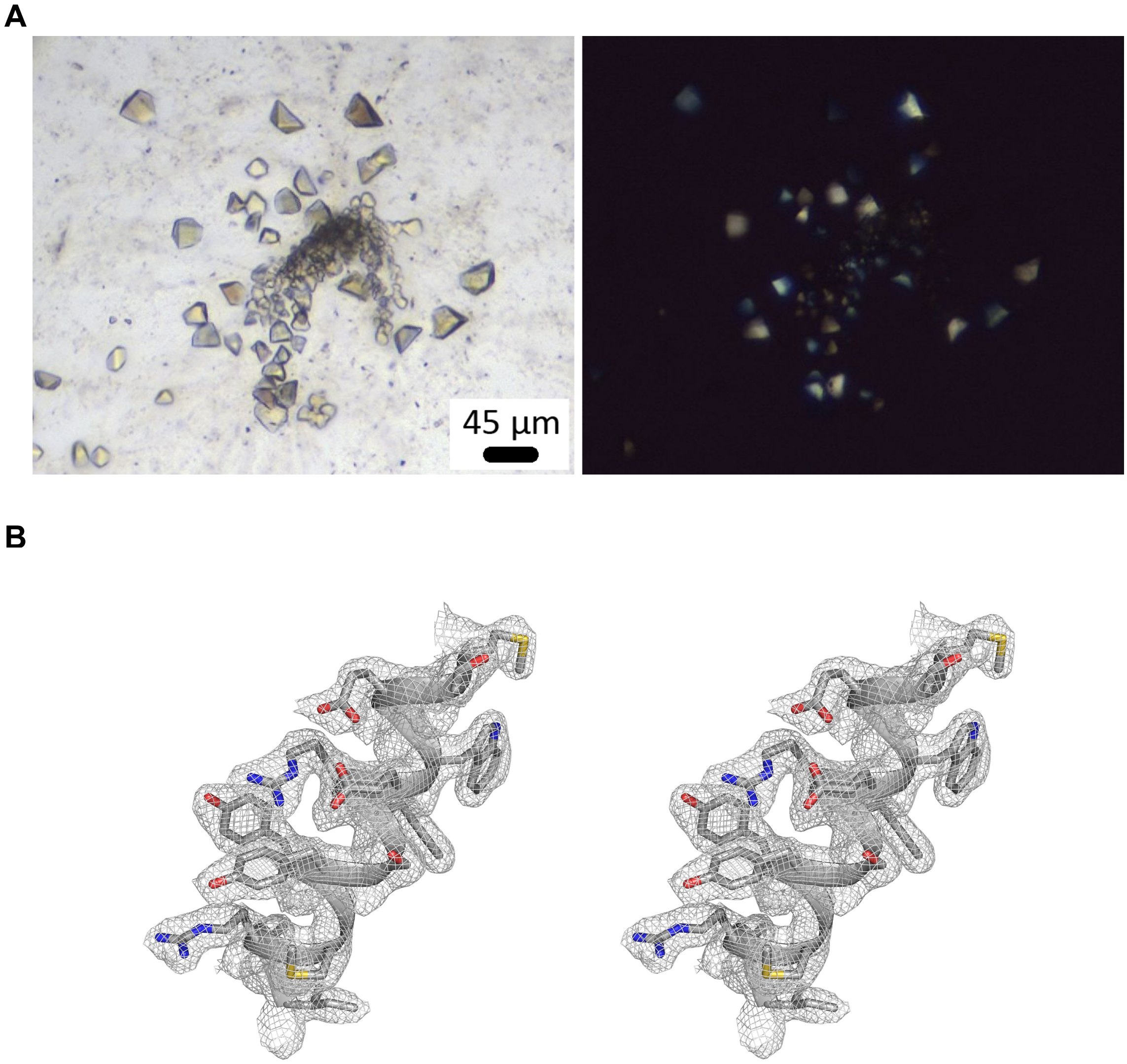

Figure S3. Crystals and electron density map of PepT$_{St}$. A. Crystal images of PepT$_{St}$ recorded after 5 days in the cubic phase under brightfield (left) and cross polarized light (right). **B.** Crystals of PepT$_{St}$ in 7.8 MAG diffracted X-ray to a maximum resolution of 2.5 Å providing high quality electron density maps. Shown in stereo view, a section of TM1 (residues 20-35) of apo PepT$_{St}$ containing the ExxERFxYY motif with *2mFo-DFc* electron density (light grey) contoured to 1 σ.
