# Supplementary material for: Structural basis for polyspecificity in the POT family of proton-coupled oligopeptide transporters
Source: EMBO Rep. 2014 Jun 10;15(8):886–93. doi: 10.15252/embr.201338403 (PMC4149780; doi:10.15252/embr.201338403)
Supplement: Supplementary file 4 [file embr0015-0886-sd4.pdf]

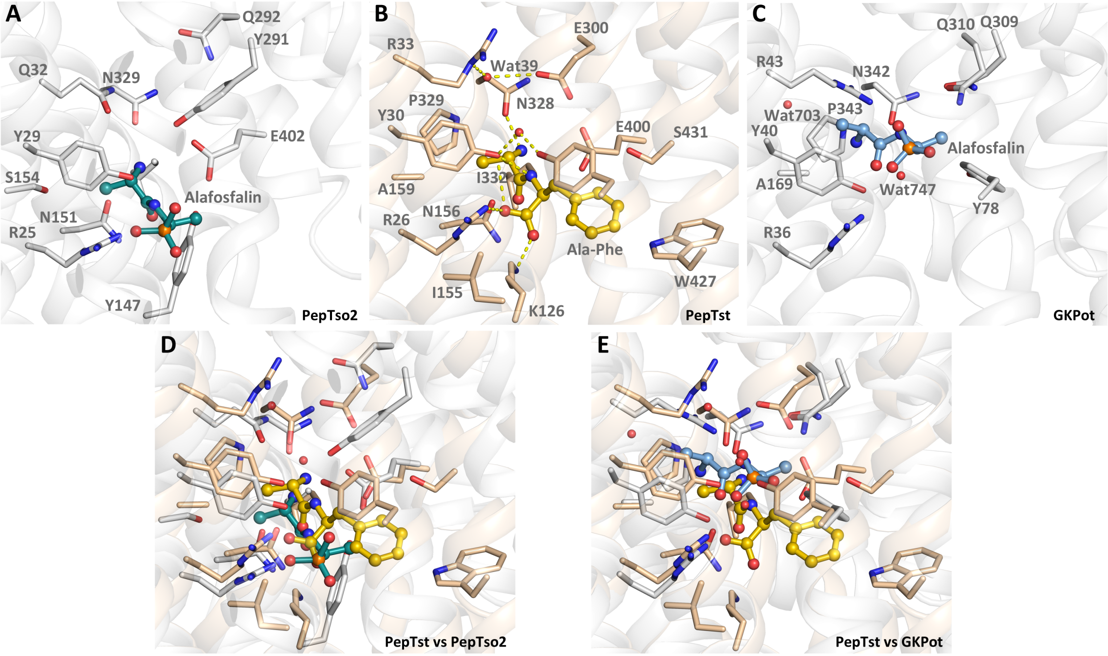

Figure S4. Comparison of the Ala-Phe binding site with published alafosfalin complexes. A. Alafosfalin binding site in PepT$_{So2}$ (PDB ID: 4LEP). **B.** Ala-Phe binding site in PepT$_{St}$ (current study). **C.** Alafosfalin binding site in GkPOT^{E310Q}:alafosfalin complex (PDB ID: 4IKZ). Ala-Phe binding site comparison with **D;** the PepT$_{So2}$:alafosfalin complex and with **E;** the GkPOT$_{E310Q}$:alafosfalin complex. Alafosfalin in both GkPOT and PepT$_{So2}$ largely bind in a similar orientation to Ala-Phe (D and E). The PepT$_{So2}$:alafosfalin complex is most similar to the PepT$_{St}$:Ala-Phe complex. However, differences due to 1) the size of the phosphonate group and 2) sequence conservation in the extracellular gate (in PepT$_{So2}$ glutamates occupy the Arg33 and Glu300 loci) contribute to subtle differences in interactions with the substrate. In GkPOT$_{E310Q}$ the ligand sits deeper into the protein disrupting the extracellular salt bridge (water mediated between Arg33 and Glu300 in PepT$_{St}$ (B)) between Arg43 and Gln310, where Arg43 moves to interact with the phosphonate. Unlike the Ala-Phe-PepT$_{St}$ complex, the homologs of Arg26 and Lys126 do not interact directly with the phosphonate. However, Arg43 interacts via a long hydrogen bond to Wat747. The hydrophobic pocket is only present in the Ala-Phe complex. Binding sites

(chosen within 4 Å of the respective ligands with addition of extracellular gates) in $PepT_{St}$, $PepT_{So2}$ and GkPOT are shown. Ala-Phe, $alafosfalin_{PepTso2}$ and $alafosfalin_{GkPOT}$ are shown in ball and stick with yellow, teal and blue carbons respectively. Hydrogen bonds and electrostatic interactions are shown with yellow dashed lines.
