# Supplementary material for: Structural basis for polyspecificity in the POT family of proton-coupled oligopeptide transporters
Source: EMBO Rep. 2014 Jun 10;15(8):886–93. doi: 10.15252/embr.201338403 (PMC4149780; doi:10.15252/embr.201338403)
Supplement: Supplementary file 5 [file embr0015-0886-sd5.pdf]

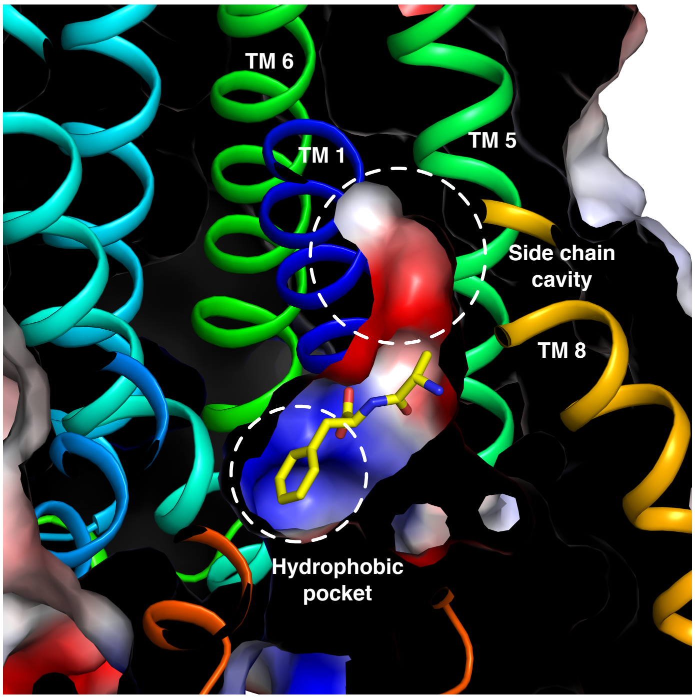

Figure S5. A cavity exists in PepT$_{St}$ above the alanine side chain of the di-peptide ligand that would comfortably accomodate larger side chain groups. Side view of the Ala-Phe complex showing the position of the di-peptide in the binding pocket and the Van der Waals electrostatic surface of the protein. The hydrophobic pocket discussed in the main text is highlighted for orientation, and the additional cavity opening up above the alanine side chain is emphasized. This is constructed largely from side chains contributed by H1 and H5 from the N-terminal bundle and H7 and H8 from the opposing C-terminal bundle.
