# Supplementary material for: Structural basis for polyspecificity in the POT family of proton-coupled oligopeptide transporters
Source: EMBO Rep. 2014 Jun 10;15(8):886–93. doi: 10.15252/embr.201338403 (PMC4149780; doi:10.15252/embr.201338403)
Supplement: Supplementary file 6 [file embr0015-0886-sd6.pdf]

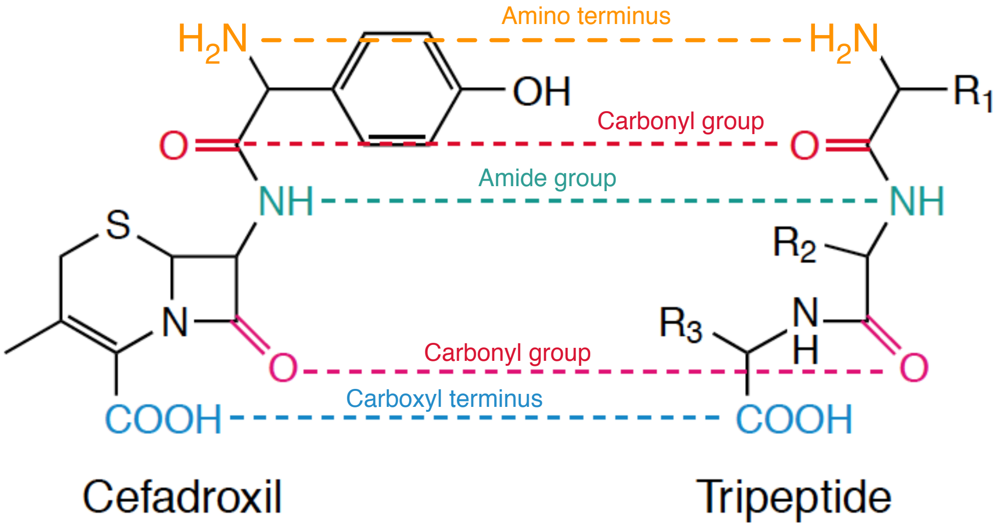


**Figure S6. Structural similarity between β-lactam antibiotics and naturally occurring tri-peptides**. Cefadroxil, a well-known β-lactam antibiotic is recognized by many POT family transporters, including PepT1, whose ability to transport this drug across the intestinal brush border membrane is an important factor in the favorable bioavailability of this drug molecule. The minimal structural similarity between cefadroxil and a generalized tri-peptide is shown. Note that the R1 group in Cefadroxil is identical to the side chain of tyrosine. Figure adapted from [1].

1. Rubio-Aliaga I, Daniel H (2002) Mammalian peptide transporters as targets for drug delivery. *Trends Pharmacol Sci* **23**: 434–440.
