# Supplementary material for: Structural basis for polyspecificity in the POT family of proton-coupled oligopeptide transporters
Source: EMBO Rep. 2014 Jun 10;15(8):886–93. doi: 10.15252/embr.201338403 (PMC4149780; doi:10.15252/embr.201338403)
Supplement: Supplementary file 7 [file embr0015-0886-sd7.pdf]

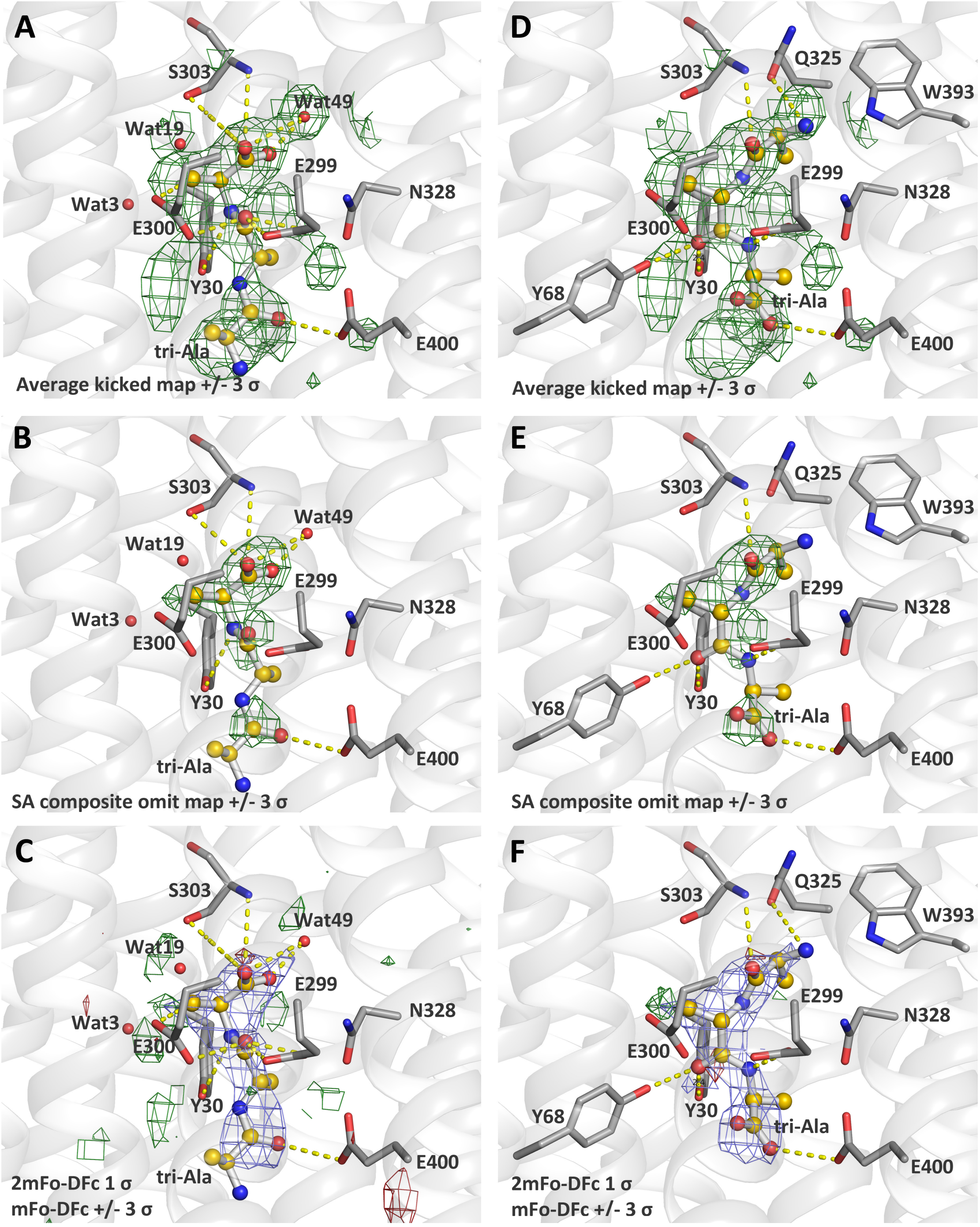

Figure S7. Comparision of two different orientations for the tri-Ala bound to PepT$_{St}$ with various electron density maps to validate the modeling. The tri-Ala peptide is modeled with the C-terminus (**A-C**) and N-terminus (**D-F**) facing the apex of the binding site cavity. Average kicked maps (**A, D**), simulated annealing composite omit maps (B, E) and mFo-DFc (**C, F**) are contoured to +/-3.0 σ (green and red, respectively). Refinement 2*mFo-DFc* maps are shown in blue and contoured to 1.0 σ. Both binding modes can

be adequately described by the average kicked and omit maps. Protein is displayed as transparent cartoon. Ligands as ball and stick with interacting residues as sticks. Polar contacts are indicated with yellow dashed lines.
