# Supplementary material for: Structural basis for polyspecificity in the POT family of proton-coupled oligopeptide transporters
Source: EMBO Rep. 2014 Jun 10;15(8):886–93. doi: 10.15252/embr.201338403 (PMC4149780; doi:10.15252/embr.201338403)
Supplement: Supplementary file 8 [file embr0015-0886-sd8.pdf]

EMBO
reports

Manuscript EMBO-2013-38403

Structural basis for polyspecificity in the POT family of proton coupled oligopeptide transporters.

Joseph A. Lyons, Joanne L. Parker, Nicolae Solcan, Alette Brinth, Dianfan Li, Syed T. A. Shah, Martin Caffrey and Simon Newstead

Corresponding author: Simon Newstead, University of Oxford

Review timeline:

Submission date:	21 December 2013
Editorial Decision:	07 January 2014
Appeal:	07 January 2014
Editorial Decision:	31 January 2014
Revision received:	22 April 2014
Accepted:	12 May 2014

Transaction Report:

(Note: With the exception of the correction of typographical or spelling errors that could be a source of ambiguity, letters and reports are not edited. The original formatting of letters and referee reports may not be reflected in this compilation.)

Editor: Barbara Pauly

1st Editorial Decision 07 January 2014

Thank you for the submission of your research manuscript to our editorial office. First of all I would like to apologize for the delay in getting back to you with a decision on your manuscript. This delay was due to the holiday season and the high number of submissions we have received during this period. I have now had the opportunity to carefully read your study and to discuss its suitability for publication with my colleagues. I regret to say that the outcome of this process is not a positive one and that we cannot offer to publish your manuscript.

We acknowledge that you have co-crystallized bacterial proton dependent oligopeptide transporters (POTs) together with di- and tri-peptides. Your structural analysis shows that while the di-peptides bind to the transporter in a horizontal way, the tri-peptides bind vertically and with lower affinity to the same binding site. We appreciate that these findings explain how the same transporter/binding site can accommodate different ligands. However, if one looks at the study from a broader perspective, we do not feel that the details of the binding mode of the two different ligands, while certainly being of interest to specialists in the field, would be of sufficient general interest to our wide readership and thus make the study suitable for consideration here.

I am providing you with an editorial decision so that you can submit your manuscript elsewhere without further delay. Please note that we publish only a small percentage of the many manuscripts submitted to us, and we can therefore only subject to external review those that have a good chance

of faring well with our reviewers and readers. I am sorry to disappoint you on this occasion and hope that this will not prevent you from considering EMBO reports for publication of your work in the future.

Appeal 07 January 2014

Thank you for your email, although clearly we are disappointed. Before I re-submit the study elsewhere I wanted to explain why this study does actually address one of the fundamental questions in the field of transporter biology and would appeal to the general readership of your journal. As you will be aware the field of membrane protein structural biology has to date focused almost entirely on the overall structure of these proteins, comparing their conformations in different states of the transport cycle. Whilst this is of course very interesting these studies have not actually advanced our understanding of how these proteins actually recognise their ligands, which from a general perspective is far more interesting.

Our study is the first to address this critical question in a system that has direct parallels with important mammalian homologues. None of the previous MFS structure/function studies can claim this level of insight, even the XylE study in Nature recently refers to XylE as a homologue of the GLUTs, but XylE doesn't actually transport glucose! We and others have convincingly shown that the bacterial POT proteins operate using the same fundamental mechanism of transport and recognition as the mammalian PepT proteins. One of the most profound questions in mammalian transporter biology is how PepT1 and PepT2 are able to transport such a board range of different ligands. Indeed this very question has recently been asked in the review by Matthias Brandsch in Current Opinion in Pharmacology (attached), which focuses entirely on PepT1 as a drug transporter. Our study explains how PepT1 is able to transport larger drug molecules in the same binding site as smaller peptide ligands. Our study is also the first to show that the MFS family actually can accommodate a polyspecific binding site. This is also of enormous general interest to the pharmacology and physiology fields. Indeed, a recent study in Nature Communications (attached) actually states "As with the examples above, the substrate multispecificity of a POT family protein is of interest in various fields of science including drug development, nutrition and fermentation". Our study shows the first structural evidence that nutrient importers operate using the same fundamental mechanism as the multi drug exporters, namely multiple binding sites.

Given the above arguments are you still sure that EMBO Reports really doesn't consider this study of general interest? PepT1 and PepT2 are enormously important transporters in human health and physiology, our study provides the definitive answer as to why the human body can assimilate peptides using only two transporters and provdes the first answer as to why beta lactam antibiotics can use pepT1 for entry into the body, surely this is interesting.

2nd Editorial Decision 31 January 2014

Thank you very much for the submission of your research manuscript to our editorial office. We have now received the full set of reviews on your manuscript.

As the detailed reports are pasted below I will only repeat the main points here. You will see that all reviewers appreciate the interest of your findings and are, in principle, supportive of publication of your study in our journal. However, they also point out aspects of your study that should be further strengthened before publication. For example, both referees 3 and 4 feel that your findings should be backed up by additional functional assays; referee 3 raises this point with regard to the question whether the tripeptides are indeed all transported and reviewer 4 makes a more general comment on this issue. This referee also states that composite omit maps should be provided and reviewer 1 asks whether the vertical orientation of one of the tripeptides might be due to the lack of a phenylalanine at its C-terminus.

Given the reviewers' constructive comments on how to improve the study, I would like to give you the opportunity to revise your manuscript, with the understanding that the main concerns of the reviewers should be addressed. Acceptance of the manuscript will depend on a positive outcome of a second round of review and I should also remind you that it is EMBO reports policy to allow a single round of revision only and that therefore, acceptance or rejection of the manuscript will depend on the completeness of your responses included in the next, final version of the manuscript.

I look forward to seeing a revised form of your manuscript when it is ready. Should you in the meantime have any questions, please do not hesitate to contact me.

Referee #1:

The authors present three crystal structures of a bacterial proton-coupled peptide transporter (apo and in complex with a di- and tri-peptide). These structures are the foundation to explain the ligand promiscuity of these transporters. Most importantly, the di- and tri-peptides are bound tot the transporter is different modes. In the case of the di-peptide a 'horizontal' binding arrangement is observed, while a 'vertical' one is determined for the tri-peptide complex. The conclusions are based on high-resolution structures that were obtained in lipidic meso phases.

Overall the manuscript is well written and most if not all conclusions are justified. In principle, I would recommend acceptance of the manuscript, although some points have to be addressed before I can make a final decision.

Below I list my points in the order of appearance and not in the order of importance
• Figure S1: here a sequence alignment is presented, but in the manuscript (page 2, 2nd paragraph the authors state that Figure S1 shows the N- and C-terminal bundle of the structure.
• Table 1 Ramachandran statistics should be added.
• Figure 1: the authors should comment why the phenyl side chain is not covered by electron density. I understand that the cut-off was 3 sigma. Nevertheless, it is surprising to see this lack of density, despite the fact that the side chain is apparently located in a deep hydrophobic cavity. How sure are the authors about the identity of this side chain?
• Page 3, tri-peptide paragraph: why was tri-alanine used as a tri-peptide and not a tri-peptide that also contained a Phe at the C-terminus? The phenyl side chain does bind in a hydrophobic pocket in the structure of the di-peptide complex and I wonder whether the orientation of the tri-peptide is influenced by the absence of Phe. In other words - is the vertical orientation a consequence of the missing hydrophobic moiety? Especially in light of the differences in affinity between AF (21 µM) and AAA (400 µM).
• Figure 2A: if I understand the Figure correctly, Glu400 is 3.3 Å away from the N-terminus of the tri-peptide, although the black, dashed line is missing. Why do the authors state on page 3 that this interaction is missing?
• I would suggest that the authors add a brief statement about peptide specificity of the transporter. This would be extremely helpful for the reader.
• Page 5, section "structural basis ..": why is an interaction between a deprotonated glutamate and a serine energetically unfavorable? These two amino acids would form a hydrogen bond?!
• Although presented in Table 1, the apo structure is not mentioned in the manuscript. If this structure is not important, it can be removed from the Table. If it is important, it should be discussed in a revised version.
• Figure S3: please change dark blue in part c to something else. Currently the difference between light and dark blue is extremely hard to see.

Referee #2:

The paper by Newstead and collaborators presents crystal structures of di- and tri-peptide bound complexes of a bacterial homologue of PepT1. The structures provide an interesting and beautiful example of a strategy to bind multiple ligands within a single binding site and emphasize the remarkable versatility of multidrug binding pockets.

I am missing a more general and thorough discussion of the literature on this question so that the findings described here can be understood in the proper context. I am not sure what is meant in the Introduction regarding the multidrug efflux systems and I think the references provided (5-7) are outdated and not appropriate for the topic discussed. Extensive work from many laboratories has contributed to our understanding of the question of promiscuity of the binding site. Work on soluble transcription factors, mainly from Brennan's lab (e.g. (1)), on the bacterial AcrAB-TolC complex (e.g. (2)), on MATE proteins (e.g. (3)) provide us with similar snapshots: in each case various ligands were shown to interact with different determinants in a unique binding cavity. The authors should discuss what is novel and different in their structures.

A minor comment: In the Introduction, page 2: "Recent crystal structures... have revealed that the POT family belongs to the MFS.... "
I do not think we had to wait for the crystal structures to know that the POT family belongs to the MFS superfamily.

1. Peters, K. M., Brooks, B. E., Schumacher, M. A., Skurray, R. A., Brennan, R. G., and Brown, M. H. (2011) A single acidic residue can guide binding site selection but does not govern QacR cationic-drug affinity. PLoS One 6, e15974
2. Nakashima, R., Sakurai, K., Yamasaki, S., Hayashi, K., Nagata, C., Hoshino, K., Onodera, Y., Nishino, K., and Yamaguchi, A. (2013) Structural basis for the inhibition of bacterial multidrug exporters. Nature 500, 102-106
3. Lu, M., Symersky, J., Radchenko, M., Koide, A., Guo, Y., Nie, R., and Koide, S. (2013) Structures of a Na+-coupled, substrate-bound MATE multidrug transporter. Proc Natl Acad Sci U S A 110, 2099-2104

Referee #3:

Lyons et al report structural data from crystal structures of a bacterial peptide transporter in complex with a dipeptide and a tripeptide. The two peptides bind in partially overlapping sites but in very different manner and orientation. As a structural study it is methodologically excellent, well-written, brief and to the point. It is a significant contribution to understanding peptide binding to peptide transporters that in itself would be of sufficient quality and relevance to the readership of EMBO Reports. However, the authors make the somewhat bold inference that the difference in binding orientation that they observe can also explain how the bacterial transporters can accommodate different substrates, i.e. it reveals the basis for polyspecificity in transport. Unfortunately, several places in the manuscript they fail to clearly state whether they are describing polyspecificity in transport or just polyspecificity in binding. This ambiguity is a major issue because the most interesting but also challenging study would be a description of how polyspecificity is achieved in transport, yet their data predominately allow them to conclude on differences in binding. It remains unclear and in my opinion unlikely that the extended tripeptide could be translocated from the more distal site where it is found in the crystal. Instead, this site may be a high-affinity transit site for the tripeptide on its way to a low-affinity site akin to the deeper dipeptide site from where the tripeptide can eventually be translocated across the membrane. If the authors want to conclude only on differences in preferred binding modes for dipeptide compared to tripeptides it should be more clear in the title and in the manuscript. If they believe that the basis for polyspecificity in transport is implied by their data it should be much more clearly stated that this is to some extent a speculation based on the structural data and that there is limited functional data to support the alternative transport mode described in Figure 4. Alternatively, if the strong wording is to be maintained my suggestion would be to include some functional data that would support that the tripeptide is indeed translocated from the site found in the crystal, for example by kinetic data that shows that transport rates and/or apparent affinities for tripeptides are much less affected by mutations around Pocket 2 than the transport rates and/or apparent affinities for the dipeptide. It is difficult to judge solely from the figures but for example N328Q may be a subtle mutation that would still be sufficient to provide functional data to support that the authors claim regarding the basis for polyspecificity could also be extended to polyspecifictiy in transport.

Minor issues:
-The authors should at least discuss whether tri-Ala peptide is the most relevant tripeptide representative to us in a comparison with Ala-Phe. Why is Phe-Ala-Ala or Ala-Phe-Ala not used?
-The explanation for Cefadroxil binding and transport may be correct but is speculative and only very weakly supported by the experimental data and primarily based in an automated in silico docking. This should be stated more clearly in the main article and separated from the experimental data regarding tripeptide binding (p4, middle).

Referee #4:

In the manuscript "Structural basis for polyspecificity in the POT family of proton coupled oligopeptide transporters', Lyons et al. report two crystal structures of a bacterial peptide transporter protein PepT1 complex with di- or tri- peptide ligands. They observed different ligand binding conformations in the central cavity of the protein. Although three structures of POT protein bound with different ligands have been recently published (ref. 13-15) and one was from the authors' lab, these two new POT complex structures may provide additional insight into the mechanisms of substrate recognition and transport. The manuscript is interesting and well written. However, a few of concerns, particularly regarding the ligand model, need to be addressed before publication.

Major issues:

1. To confirm the ligand conformations, it would be important to calculate and show composite omit map instead of fo-fc different map in Fig. 1A and 2A. The fo-fc map may not completely eliminate the model bias, although the structures look reasonably refined. The tri-Ala ligand conformation may be an issue. With the map shown in Fig. 2A, it is ambiguous to dock the ligand as the conformation claimed by the authors. E.g. the ligand may flip over with similar fitting and its terminal amino group would perfectly form a salt-bridge with the carboxylate group of the conserved residue E300. Careful confirmation of the ligand binding conformations is essential since the authors discuss ligand interactions at atomic level in a great deal.

2. Lack of any functional assay is another weak side of the manuscript. E.g. in pg. 3 last paragraph, R26-E400 absent in the tri-Ala peptide interaction may explain its weaker binding affinity. A simple binding assay of the double mutant with the two ligands will test this hypothesis and may help to confirm the different ligand binding conformations as questioned above.

3. The authors need to explain any overall structural difference between their occluded conformation and other published POT structures, which could be important for the transport mechanism.

4. In pg 4 last paragraph, the authors discuss discrete pockets in the binding site. It is hard to imagine the subtle difference from the two separated views. The authors need additional figure to show superimposition of the two binding sites with stereo view here.

Minor issues:
1. pg 5 line 6, if it is noticeably compact, show rmsd value.
2. Pg 5 paragraph 3 line 5, no TM10 in Fig. 3B.
3. Table 1, show average B value of the protein and ligands
4. Fig. S3, stero view.

1st Revision - authors' response 22 April 2014

We were very pleased that the referees found our study titled '**Structural basis for polyspecificity in the POT family of proton coupled oligopeptide transporters**' well written with most, if not all, the conclusions justified. In particular the referees thought the structures provide 'an interesting and

beautiful example of a strategy to transport multiple ligands within a single binding site'. In addition the referees recognize that the paper is 'a significant contribution to understanding peptide binding to peptide transporters', with the crystal structures alone of 'sufficient quality and relevance' to those working on membrane transporters and more generally the scientific community served by EMBO Reports. The referees suggested that the study could be strengthened further. Specifically if **additional** biochemical data were presented that supported our original hypothesis at the functional level for the 'multi-mode' binding model. As we have detailed below we have included the requested functional data and are confident the new information **supports our original model** and **strengthens the overall study** such that we would like this revised manuscript to be considered for publication in EMBO Reports.

Addition of new functional data to support the multi-mode binding model.

As pointed out in the initial decision letter, Referees 3 & 4 felt that the multi-mode transport model, which was based on the structural data presented in our first draft, should be supported by functional studies. We accept this would strengthen the study and have undertaken a series of detailed kinetic studies using reconstituted $PepT_{St}$ in liposomes. Figure 2C shows the effect of binding site mutants on proton driven uptake of the WT protein and mutants using a [^{3}H]-L-Ala-L-Ala peptide uptake assay we developed in our group for the initial functional study of $PepT_{St}$ published in EMBO J in 2012 (Solcan *et al.*, EMBO J 31, 3411-21 2012). Some of these mutants were previously described by our group [1], however we have included the data here in addition to other novel mutants to illustrate the importance of these residues on peptide uptake in $PepT_{St}$. Figure 2D and 2E show competition data to determine the effect of interfering with key binding site side chains on IC_{50} values. The results of these studies provide evidence that the di-Ala peptide and tri-Ala peptide interact with different side chains within the binding site. In the revised manuscript we focused on two key questions raised by the referees. In particular we wished to determine if specific mutations in the binding site could affect di- and tri-peptide transport differently, providing further evidence that peptides can be recognized differently within the binding site. Our results, shown in Figure 2, panels C-E, reveal that indeed we can identify specific residues that affect di and tri-peptide transport; even more convincing is that these mutations were selected based on the complex structures. For example, we observed in the Ala-Phe complex that Asn156 makes a H-bond interaction with the carbonyl oxygen (Figure 1B) but does not interact with the modeled tri-alanine (Figure 2A). Subsequent analysis of the N156A mutant revealed that only di-peptide transport was affected, with a small reduction in the IC50 value (Figure 2D) but no effect was observed in the tri-alanine assay (Figure 2E). Likewise, we could identify mutations that also only affected tri-alanine transport, such as Y29F ad W427F.

These results provide additional evidence that these peptides **do interact differently within the binding site of $PepT_{St}$**. We appreciate that ideally we would have liked to measure the effect of the Y30F mutant on tri-Ala transport, as this residue is observed interacting with the tri-Ala peptide

© European Molecular Biology Organization

density. However, Y30F was severely reduced in overall uptake (Figure 2C), complicating interpretation of any kinetic measurements made on this mutant.

We conclude from this study that peptides do interact differently within the binding site, as per our original model, but that the interactions are not entirely independent of one another. Indeed, we have amended our discussion section to suggest that our data presents a likely scenario that the binding site is able to accommodate peptides in a dynamic way, adapting to the different side chain chemistries as necessary. In light of this change in emphasis we have also altered our final transport model, Figure 4, to suggest that at least two modes of transport are possible; a vertical mode and a lateral mode, depending on how best to accommodate the particular peptide being transported. Although as we now state in our discussion section, in our opinion it is highly likely that additional binding modes exist and that our study reveals two of possibly multiple orientations that peptides might adopt during binding and transport.

Our re-analysis of the tri-peptide complex data.

To improve the rigor of our analysis of the tri-peptide complex we have extensively re-analyzed the electron density maps for the tri-peptide complex, including the calculation of simulated annealing composite OMIT and averaged kicked maps (Figure S7 and Figure 2A). We have detailed this analysis in the Supplementary Information.

Our conclusion from this analysis is that the tri-alanine is likely to exist in the crystals with reduced occupancy and held less tightly than the di-peptide. This is consistent with the IC_{50} values determined for tri-alanine competition with di-alanine of 0.4 mM, the elevated atomic displacement factors for tri-alanine in the crystal structure (Table 1) and also the noticeably less compact structure $PepT_{St}$ adopts in the tri-Ala complex vs. the di-Ala complex (Figure 3A). Nevertheless, the resulting electron density maps clearly show the tri-peptide adopts a vertical orientation (Figure 3A and subsequent analysis in SI Figure S7). We have modeled the peptide with its C-terminus facing the extracellular side of the binding site based on previous functional data from rabbit PepT1 suggesting the C-terminus of the peptide interacts with residues at the extracellular entrance to the binding site [2]. However, the electron density maps do not provide unambiguous information to orient the tri-Ala peptide with respect to the N- or C-terminus and we acknowledge that it is very possible the tri-peptide may exist in our crystal structure as a mixed population. To be absolutely rigorous in our presentation of this data we have acknowledged this caveat in the main text and further added a description of this in the PDB header.

We feel that the additional functional data and careful re-analysis and representation of the crystallographic data in light of the referee's comments address the main concerns with the original submission. Below we have addressed specific points raised by the referee's:

Referee #1 (Remarks to the Author):

1. *Figure 1: the authors should comment on why the phenyl side chain is not covered by the electron density.*

The pocket for the phenyl side chain is large and the side chain is distant from most other residues - closest contact is 3.5 Å to Trp427, which itself is not well resolved in the maps. Our sense is that there is rotamer flexibility in the phenyl side chain to a small degree that can explain the disorder.

Separately, we have confirmed the identity of the di-peptide we used in this study using NMR. The spectra confirm that the peptide is L-Ala-L-Phe. We are confident therefore of the current modeling and interpretation.

2. *Page 3, tri-peptide paragraph: why was tri-alanine used as a tri-peptide and not a tri-peptide that also contained a Phe at the C-terminus? "Is the vertical orientation a consequence of the missing hydrophobic moiety?"*

Our new functional data, which we reported in Figure S2 (Supplementary Information), indicates that $PepT_{St}$ preferentially recognizes tri-alanine over other tri-peptides, as originally reported in our 2012 study of $PepT_{St}$ [1]. Our complex with tri-alanine therefore most likely represents the most functionally relevant snapshot of how longer peptides interact with the binding site in this transporter.

However, we agree with the referee that we should not dismiss the possibility that other tri-peptides may adopt different conformations. In light of these comments, and those from Referee 3 (below), we have toned down our more rigid model that di-peptides binding laterally and tri-peptides bind vertically. We have now modified the text to suggest that our structural data show that peptides can interact within the binding site in different modes and that these are not necessarily restricted to peptide length alone. However, in our new functional data investigating the affect of point mutations in the binding site (Figure 2D & 2E) we show that certain amino acids (N156A) decrease the IC_{50} for Ala-Ala, while having no affect on the IC_{50} for tri-Ala, while others (Y29F & W427F) modulate the IC_{50} for tri-Ala and have no discernable affect on di-Ala.

We are confident therefore that our proposed multi-modal mechanism for binding is correct, but accept that our present data does not provide sufficient information to support our earlier statement that this is di- or tri-peptide specific. Indeed, we cannot rule out that some tri-peptides could bind laterally and some di-peptides vertically.

© European Molecular Biology Organization

3. *Figure 2A. "Is Glu400 interacting with the N-terminus of the modeled tri-Ala peptide?"*

In our modeled structure of the tri-alanine complex the side chain of Glu400 sits ~ 5 Å away from the amino group of the tri-alanine peptide. However, Glu400 does sits within H-bond distance of the carbonyl group of the first peptide bond, suggesting that a functionally important interaction between the N-terminus of the peptide and Glu400 may occur during transport. Functional data presented in Figure 2C shows that mutating Glu400 to alanine abolishes di-peptide transport and equivalent glutamates in all functionally characterised POT/PTR transporters to date have shown this residue is both absolutely conserved and essential for transport. However the exact role played by this residue in the transport mechanism remains unclear. Our current structures provide further evidence of a role in interacting with the peptides, but whether this is for recognition or to trigger conformational changes is less clear.

4. *I would suggest that the authors make a brief statement about peptide specificity. This would be extremely helpful for the reader".*

Referee 2 below also raised this important point. We are conscious of the word limit for an *EMBO Report* article and feel that a more in depth discussion would not fit within the word limit. However, we have re-worded the introduction section to include the following sentences "*POT family transporters are generally regarded to transport both di- and tri-peptides* [3]. *However for* $PepT_{St}$ *and other bacterial members of the family there exists some selectivity* [4,5]. *We previously showed that* $PepT_{St}$ *displays broad specificity to di-peptides, being able to transport a range of charged and hydrophobic peptides with* IC_{50} *values in the region of 5 – 400 uM* [1]. *Tripeptides are also transported by this system, with tri-alanine being the best recognized with an* IC_{50} *value comparable to that of the di-peptides (Figure S2)."* We hope this provides a general overview of the promiscuity of the POT family as a whole and $PepT_{St}$ in particular. We have also included additional functional data to show that $PepT_{St}$ does not recognize all tri-peptides in similar way; in the small library we tested for example it much prefers tri-alanine. We would also like to highlight that our transport data for $PepT_{St}$ shows that this protein does not transport all di-peptides equally well. Indeed, tri-alanine competes more effectively than Lys-Lys for example. We are of the opinion therefore that this data further strengthens our choice of co-crystallization with tri-Ala, as a representative ligand of the larger peptides recognized by this system.

5. Page 5, section 'structural basis..": why is an interaction between a deprotonated glutamate and serine energetically unfavorable? These two amino acids should form a H-bond?!"

We agree with the referee that our description of this mechanism was not clear in the original draft. In the revised manuscript we have focused on the observed formation of the hydrophobic pocket in response to Ala-Phe binding, which our structures resolve for the first time, and suggest this as the first experimental evidence for an induced fit mechanism operating within

the POT/PTR family of transporters. We have subsequently re-titled the sub-heading "Intracellular gate linked to formation of a hydrophobic pocket" to reflect this.

6. Referee 1 suggested other typographical corrections and alternations, which we have now completed.

Referee #2 (Remarks to the Author):

1. "I am missing a more general and thorough discussion of the literature".

We appreciate the brevity with which we have covered the literature on peptide transporters in this study. However, given the word limits of the journal we felt that more space should be devoted to the results in this case. We do understand that context is important and have tried to re-draft the introduction to bring in a more general overview of peptide transporters and their promiscuity.

2. Referee 2 also suggested using different references for the multi drug exporter examples and provide a more in depth discussion comparing the two systems.

Given the space limitations and the addition of the new functional data we felt that, in hindsight the comparison with multidrug transporters was not entirely appropriate for this study and have removed it from the current version. This type of compare-and-contrast discussion is probably best left to a separate review of transport mechanisms.

3. Referee 2 made a number of minor comments and these have been addressed in the current version.

Referee #3 (Remarks to the Author):

1. Referee 3 commented that in places we had not adequately distinguished the difference between binding and transport.

We agree that our study, as first drafted, dealt largely with binding, from the structure data, and extended these observations to speculate on transport. We have tackled this issue through the functional studies we discussed at the start of this letter and which provide evidence that during transport the two peptide we tested appear to be handled differently by $PepT_{St}$. Given the supporting functional data from the point mutants, which where themselves identified through the structures of the peptide bound complexes, we believe the present revised study presents further experimental evidence for our initial multi mode model to explain the polyspecificity observed in these proteins. We acknowledge that the current submission presents a starting point for further biochemical studies into how different peptides are recognized. We have reduced the forceful tone employed in the first submission as we now believe, as discussed above, that different binding modes may be employed to recognize both di- and tri-peptides depending on the best accommodation of their side chains within the binding site.

Referee #4 (Remarks to the Author):

© European Molecular Biology Organization

Referee 4 requested additional maps to confirm ligand conformations. Figure 2A has been updated to include a sigma A weighted averaged kick map [6]. Average kick maps (or AK maps) were reported in 2009 by Paul Adams and Dusan Turk as a new way to calculate unbiased maps for model building, especially where unambiguous map interpretation is not available. They are calculated using the Phenix Crystallograpy Suite and in short they are the result of averaging a series of maps where each map is calculated from atomic coordinates modified by random shifts, i.e. a kick. In this study the 'kicks' were applied to the tri-Ala peptide and the resulting averaged map shown in Figure 2A. Analysis presented in the 2009 Acta Crystallographica D paper showed that averaged kick maps corresponded better, or at least no worse, than traditional sigma-A and simulated annealing maps.

A new Supplementary Figure S7 showing averaged kick, simulated annealing composite omit and the refined maps have been included. We agree with the referee that we cannot unambiguously differentiate between the two orientations of the tri-peptide. The orientation chosen originally was based on previous biochemical findings, as detailed at the start of this letter. The text has been reworked to this effect. On reflection, we have decided that the tri-peptide model will be deposited in a ligand free state to avoid confusion for people using these models to understand peptide recognition. We will detail this decision in the PDB header. As mentioned above, we are confident in our analysis of a vertical binding mode for tri-alanine both from a crystallographic and functional standpoint.

Functional data has been added that support our hypothesis of a multi-mode binding site – please see above.

"The authors need to explain any overall structural difference between their occluded conformation and other published POT structures, which could be important for the transport mechanism."

We have reworked the main text to detail the differences between the three structures reported and included more detailed analysis in Figure 3A-C. However, we do not see an occluded conformation of $PepT_{St}$ and apologise if this came across in the original submission. What we observe is a noticeable compaction of the structure with Ala-Phe bound vs. the tri-Ala bound or apo structures, with the main movement occurring in the C-terminal helix bundle. This movement, illustrated in Figures 3A-C, results in the formation of the hydrophobic pocket observed in the Ala-Phe structure. This leads to the logical conclusion that the transport mechanism likely has an induced fit component, as previously suggested for LacY the lactose permease from *E. coli*. Whether this hydrophobic pocket forms when peptides are transported in other orientations remains to be determined.

"In pg 4 last paragraph, the authors discuss discrete pockets in the binding site. It is hard to imagine the subtle difference from the two separated views. The authors need additional figure to show superimposition of the two binding sites with stereo view here."

Following the text rework during revision to make the discussion of pockets more succinct we do not feel this figure is now necessary.

Minor issues have been addressed.

Please accept our revised manuscript, which we sincerely hope will be accepted for publication.

References

1. Solcan N, Kwok J, Fowler PW, Cameron AD, Drew D, Iwata S, Newstead S (2012) Alternating access mechanism in the POT family of oligopeptide transporters. *EMBO J* **31**: 3411–3421.
2. Meredith D, Temple CS, Guha N, Sword CJ, Boyd CA, Collier ID, Morgan KM, Bailey PD (2000) Modified amino acids and peptides as substrates for the intestinal peptide transporter PepT1. *Eur J Biochem* **267**: 3723–3728.
3. Daniel H, Spanier B, Kottra G, Weitz D (2006) From bacteria to man: archaic proton-dependent peptide transporters at work. *Physiology (Bethesda, Md)* **21**: 93–102.
4. Prabhala BK, Aduri NG, Jensen JM, Ernst HA, Iram N, Rahman M, Mirza O (2014) New insights into the substrate specificities of proton-coupled oligopeptide transporters from E. coli by a pH sensitive assay. *FEBS Lett* **588**: 560–565.
5. Harder D, Stolz J, Casagrande F, Obrdlik P, Weitz D, Fotiadis D, Daniel H (2008) DtpB (YhiP) and DtpA (TppB, YdgR) are prototypical proton-dependent peptide transporters of Escherichia coli. *FEBS J* **275**: 3290–3298.
6. Praznikar J, Afonine PV, Guncar G, Adams PD, Turk D (2009) *Acta Crystallogr D Biol Crystallogr* **65**: 921–931

2nd Editorial Decision 05 May 2014

Many thanks for your patience while your revised study was evaluated by the referees. They have now returned their reports to our editorial office and I am happy to tell you that they now all support publication of the manuscript in our journal. Referee 3 still has a comment that I am forwarding to you for your information, but s/he nevertheless recommends publication of the study without further changes.

Formally, I would like you to let us know which statistical test you have used to calculate the standard deviations and error bars in Fig 2 C-E and in Fig S 2 and to indicate clearly how many independent times each of these experiments has been performed (biological, not technical replicates).

Also, please include the accession numbers of the structures in the PDB database in the final version of the manuscript.

The easiest way is if you send this information as email attachments (including the modified manuscript file in which you have clarified the statistical tests used (see above).

I will then upload them to the other files and proceed with the official acceptance and publication of your study.

Referee 3:

My original primary objection was that the authors fail to distinguish uptake from binding and that they implicitly equate binding affinities to uptake apparent affinities. Their results are based on binding (both structural and functional), yet they conclude on the uptake process. These confusions persist.

Although the authors present a di-Ala uptake assay they use it to measure inhibitory

potencies of di-Ala and tri-Ala. Firstly, changes in inhibitory potencies does not mean that these peptides are indeed transported with different apparent affinities, which is ideally what the authors should show to support their conclusions. Secondly, why are these inhibition studies not done with the Ala-Phe peptide that the authors build their conclusions on instead of di-Ala?

The additional functional data provided by Lyons et al does strengthen the manuscript. Especially, the inhibition data from the Y27F mutation support the author's conclusions. While I still believe that the authors conclusions may be bordering on the speculative based on their functional data and that they could have performed their assays differently to more directly address these concerns, the additional data do to some extent support the important conclusions drawn from the strong structural studies. I therefore recommend the publication of the revised study.

© European Molecular Biology Organization
